# A randomized controlled trial on the effectiveness of strength training on clinical and muscle cellular outcomes in patients with prostate cancer during androgen deprivation therapy: rationale and design

**DOI:** 10.1186/1471-2407-12-123

**Published:** 2012-03-29

**Authors:** Lene Thorsen, Tormod S Nilsen, Truls Raastad, Kerry S Courneya, Eva Skovlund, Sophie D Fosså

**Affiliations:** 1 Department of oncology, Oslo university hospital, Oslo, Norway; 2 Department of Physical Performance, Norwegian School of Sports Sciences, Norway; 3 Faculty of Physical Education and Recreation, University of Alberta, Edmonton, Canada; 4 School of Pharmacy, University of Oslo, Oslo, Norway; 5 Norwegian Institute of Public Health, Norway

**Keywords:** Strength training, Prostate cancer, Androgen deprivation therapy, Clinical and muscle cellular outcomes

## Abstract

**Background:**

Studies indicate that strength training has beneficial effects on clinical health outcomes in prostate cancer patients during androgen deprivation therapy. However, randomized controlled trials are needed to scientifically determine the effectiveness of strength training on the muscle cell level. Furthermore, close examination of the feasibility of a high-load strength training program is warranted. The Physical Exercise and Prostate Cancer (PEPC) trial is designed to determine the effectiveness of strength training on clinical and muscle cellular outcomes in non-metastatic prostate cancer patients after high-dose radiotherapy and during ongoing androgen deprivation therapy.

**Methods/design:**

Patients receiving androgen deprivation therapy for 9-36 months combined with external high-dose radiotherapy for locally advanced prostate cancer are randomized to an exercise intervention group that receives a 16 week high-load strength training program or a control group that is encouraged to maintain their habitual activity level. In both arms, androgen deprivation therapy is continued until the end of the intervention period.

Clinical outcomes are body composition (lean body mass, bone mineral density and fat mass) measured by Dual-energy X-ray Absorptiometry, serological outcomes, physical functioning (muscle strength and cardio-respiratory fitness) assessed with physical tests and psycho-social functioning (mental health, fatigue and health-related quality of life) assessed by questionnaires. Muscle cellular outcomes are a) muscle fiber size b) regulators of muscle fiber size (number of myonuclei per muscle fiber, number of satellite cells per muscle fiber, number of satellite cells and myonuclei positive for androgen receptors and proteins involved in muscle protein degradation and muscle hypertrophy) and c) regulators of muscle fiber function such as proteins involved in cellular stress and mitochondrial function. Muscle cellular outcomes are measured on muscle cross sections and muscle homogenate from muscle biopsies obtained from muscle vastus lateralis.

**Discussion:**

The findings from the PEPC trial will provide new knowledge on the effects of high-load strength training on clinical and muscle cellular outcomes in prostate cancer patients during androgen deprivation therapy.

**Trial registration:**

ClinicalTrials.gov: NCT00658229

## Background

Prostate Cancer (PC) is the most frequent diagnosed malignancy in men in Europe and North America. The risk of PC increases with age, and the median age of onset is approximately 70 years. Treatment depends on stage, histology and the serum PSA level, beside the patients' general health and age. Radiotherapy combined with androgen deprivation therapy (ADT) is used in patients with locally advanced tumors and/or those with high Gleason scores, characterized as intermediate or high-risk profiles [1]. ADT has potentially negative effects on several clinical outcomes, such as muscle atrophy [2].

Physical exercise during and after cancer treatment has been shown to be effective to reduce several negative clinical consequences followed by cancer and cancer treatment [3,4]. Compared to breast cancer patients relatively few studies in PC patients have been published [4,5]. More evidence on the efficacy of strength training on clinical outcomes in PC patients is therefore desirable. At the same time more attention should be paid to the effect of ADT on the muscle cell level because the reduced testosterone levels influence the underlying regulators of muscle mass and muscle function. Furthermore, the effects of strength training on the same regulators are of particular interest during ADT, and as far as we know they have not previously been explored in PC patients.

Ageing is associated with a reduction in muscle mass, typically by 1-2% per decade from the age of 30 to the age of 50. Thereafter the rate of muscle loss increases progressively to 10% per decade [6]. It is well known that decreased levels of serum testosterone are followed by reduced muscle mass [7,8]. In PC patients treated with ADT for 6-12 months, lean body mass (LBM) has been reported to decrease by 3% [9]. This reduction may affect muscle strength markedly because muscle mass is the dominant tissue in LBM.

Loss of bone mineral density (BMD) has been observed in PC patients on ADT [10,11]. It has also been shown that ADT increases body weight and fat mass in these patients [9]. Decreased levels of testosterone and body changes during ADT, may also influence mental health, fatigue and health-related quality of life (HRQOL) in PC patients [12,13].

**Figure 1:**
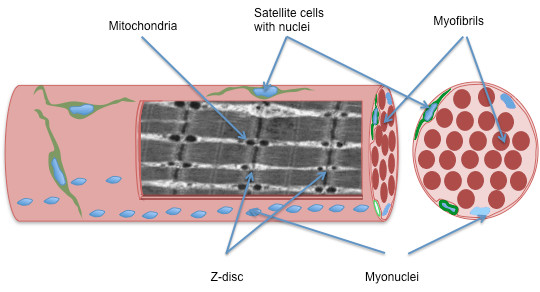
**A schematic drawing of a muscle fiber (muscle cell) in longitudinal- and cross sectional plane**. The muscle fiber is surrounded by two membranes, the ***plasma membr*ane **(inner) and the ***basal lamina ***(outer). ***The satellite cells ***are located between these two membranes, and just beneath the ***plasma membrane ***lays the ***myonuclei***. The contractile proteins in the muscle cell are arranged in ***myofibrils***. In the longitudinal plane you see that the ***myofibrils ***are organized into sarcomeres separated by the z-disc and ***the mitochondria ***are seen as circular spots between the myofibrils.

Total skeletal muscle mass reflects the size of all individual muscle groups in the body. Furthermore, the size of each muscle group is primarily determined by the size of the individual muscle fibers^1^, and to a lesser extent by the number of fibers. Regulation of muscle fiber size is first and foremost driven by an increase in the number and size of myofibrils (contractile proteins) within the muscle fiber (Figure 1). This process is normally supported by an increased number of satellite cells and (often) increased number of myonuclei (Figure 1). Importantly, satellite cells seem to play a significant role in regulation of muscle fiber size. Satellite cells are mononuclear progenitor cells that are found between the sarcolemma and the basement membrane in the muscle fibers (Figure 1 and Additional file 1). Satellite cells are normally in a quiescent state, but are activated by adequate stimuli (e.g. strength training). Upon activation the satellite cells donate their nuclei to the existing muscle fibers and thus facilitate skeletal muscle hypertrophy by increasing the protein synthesis [14]. During regeneration after muscle injury, satellite cells may also fuse and thereby form new mature muscle fibers [14]. In addition, satellite cells seem to support muscle hypertrophy by production of local growth factors (e.g. IGF-1) [15].

In the muscle tissue, testosterone stimulates both the muscle protein synthesis in muscle fibers and activation of satellite cells through the interaction with the androgen receptor [16]. Consequently, graded dosage of testosterone has been shown to both increase the muscle fiber size, reflected by increased muscle fiber cross sectional area, and number of satellite cells in a dose dependent pattern [17,18]. Suppression of testosterone reduces the positive stimuli on muscle protein synthesis, and patients treated with ADT experience reduced LBM [19,20]. Low levels of testosterone might also influence the regulation of muscle mass through its inhibition of the ubiquitin-proteasome system [21]. Consequently, ADT might facilitate muscle atrophy both by inhibiting important pathways involved in muscle hypertrophy, as well as stimulating pathways involved in protein degradation.

Muscle strength is mainly determined by the size of muscles, but other qualities of muscles, such as endurance and stress tolerance, are more related to the content and function of stress proteins and proteins involved in the mitochondrial function [22,23]. Interestingly, castration has been shown not only to reduce muscle fiber size, but also to affect the mitochondrial structure negatively in rat muscles [24]. Furthermore, castration seems to reduce the stress induced up-regulation of stress proteins in heart muscle [25]. Whether castration also affects the stress protein response and mitochondrial function in human skeletal muscles is currently not known.

### Effects of strength training

Strength training has the potential to increase muscle mass and BMD in both young and elderly males and females [26-29]. Although the response to strength training differs among individuals, most studies report an increase in muscle fibre cross sectional area by 10-60% over 9-30 weeks of training [30]. Furthermore, an increase in BMD from 1 to 4% has been observed over 16-52 weeks of strength training [27,31-33].

Effectiveness of exercise on clinical outcomes has been reported in numerous studies in cancer patients. Research has so far shown promising effects on among others muscle strength, aerobic fitness, fatigue, anxiety and quality of life [3,4]. However, the effect of exercise during ADT in PC patients has been less studied [4,5]. These studies have shown positive results in muscular and aerobic endurance, fatigue and QoL [5,34,35]. Less promising is the effect of physical exercise on body-composition endpoints. It could therefore be hypothesized that ADT compromises some important cellular signals regulating the exercise induced increase in lean body mass with strength training.

**Figure 2:**
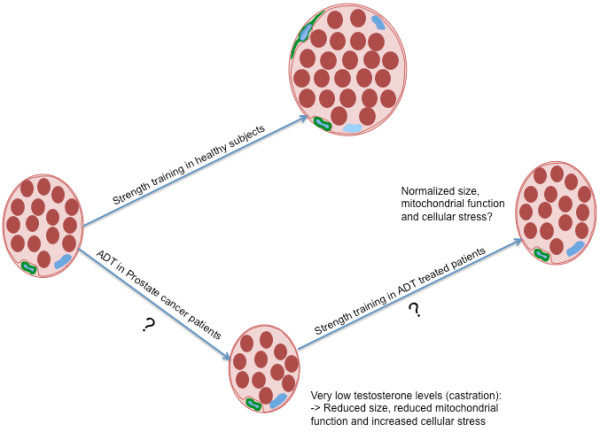
**Schematic muscular adaptations to strength training in healthy men and PC patients on ADT**. Schematic muscular adaptations to strength training in healthy men (**A**), possible consequences of ADT on muscle fibers in PC patients (**B**), and possible muscular adaptations to strength training in PC patients on ADT (**C**). In A), the muscle fiber cross sectional area is increased as a result of an increase in the number and size of myofibrils within the muscle fiber, and this increase in size is supported by an increased number of satellite cells and (often) increased number of myonuclei. In B), ADT results in decreased muscle fiber cross sectional area and reduced muscle function. In C), muscle fiber cross sectional area and muscle function is normalized in ADT treated PC patients on strength training.

The increase in muscle fiber size in response to strength training seems to be dependent, at least to some extent, on satellite cell activation in order to incorporate new myonuclei in the growing muscle fiber [14]. In healthy subjects, strength training increases the number of myonuclei and the number of satellite cells in both young and elderly individuals [36,37] (Figure 2). The mechanisms behind the activation of satellite cells during strength training in humans are not fully understood, but changes in local growth factors and testosterone interactions seem to be important [38]. Whereas reduced levels of testosterone during ADT negatively affect the regulation of muscle mass, strength training might counteract these detrimental effects on muscle fibres (Figure 2). On the other hand, it might be that the very low testosterone levels, comparable with castration, blunt the effects of strength training in these patients. Interestingly, healthy young men treated with a GnRH analog over 12 weeks showed the same increase in mRNA of important local growth factors in response to strength training as placebo treated controls [39]. Nevertheless, the accumulation of muscle mass during the strength training intervention was reduced in the testosterone-suppressed group compared to the placebo treated controls [40].

The effects of strength training on mitochondrial function and protection against cellular stress in muscle fibers are less studied than the effect on muscle size. Nevertheless, in previously untrained healthy men strength training has been observed to positively affect both mitochondrial proteins and stress proteins [41,42]. Consequently, strength training has the potential to counteract several negative effects of ADT on muscle size and function.

To our knowledge no previous studies have investigated the effect of strength training on the regulation of muscle size and of muscular function on a cellular level in PC patients during ADT. Furthermore, previous clinical data need to be confirmed. Here, we present the design and methods of an ongoing trial called the Physical Exercise and Prostate Cancer (PEPC) trial, which aims to explore the effects of strength training on clinical outcomes as well as explore mechanisms of the effect on muscle cellular outcomes in patients with PC during ongoing (neo)-adjuvant ADT.

### Aims

The overall aims of the PEPC trial are to evaluate the effectiveness of a high-load 16 week strength training program on a) clinical outcomes including serological parameters such as lipid profile (low and high density lipoprotein cholesterol) and low grade inflammation (C-reactive protein (CRP)) b) muscle cellular outcomes as muscle fiber size, regulators of muscle fiber size and regulators of muscle fiber function and c) feasibility of a high-load strength training program in PC patients who at intervention start have discontinued their high-dose radiotherapy and are on ongoing (neo)-adjuvant ADT throughout the intervention period.

## Methods/design

This study is a randomized clinical trial with two arms comparing an exercise intervention group (EG) that receives a 16 week high-load strength training program with a control group (CG) that is encouraged to maintain their habitual activity level and not start strength training. The study has been approved by the Regional Committee for Medical and Health Research Ethics, South-East Region (protocol nr. 08/212b.2008/4062).

### Participants

Patients are included from two oncological units at Oslo University hospital, the Norwegian Radium Hospital (NRH) and Ullevaal University Hospital (UUH). All patients must fulfill the following eligibility criteria 1) PC cancer of intermediate or high-risk profiles, 2) high-dose radiotherapy with or without two high-dose-rate brachytherapy fractions [43], 3) (neo)-adjuvant ADT by commercially available LHRH analogue for 9-36 months. The most important inclusion and exclusion criteria are 1) less than one hour car drive between the patient's residence and the place of training or 2) current regular strength training. All inclusion and exclusion criteria are listed in Additional file 2.

**Figure 3:**
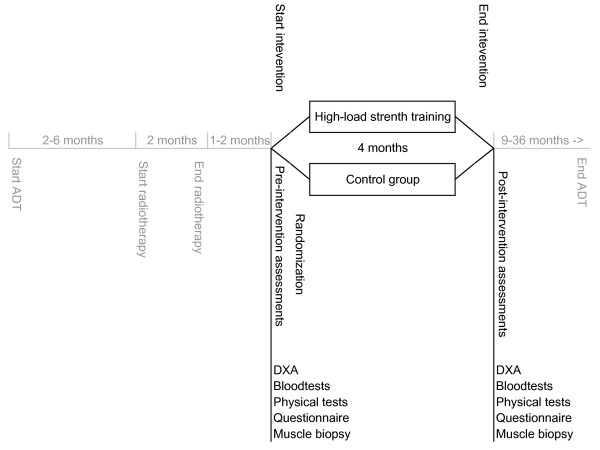
**Timeline in the PEPC trial**. Different duration of ADT related to different risk groups and somehow different treatment strategies in the two hospitals. Importantly all patients are on ADT at pre- and post-intervention assessments.

In addition to written information, eligible patients are verbally informed about the study by their responsible radiotherapist and the study coordinator usually during or immediately after radiotherapy. After giving their written consent agreeing patients are scheduled for pre-intervention assessments usually 1-2 months after radiotherapy, which are repeated one week after the intervention period (Figure 3).

### Randomization

At completion of the pre-intervention assessments patients are randomized in a 1:1 ratio to the EG or CG, stratified for hospital. Randomization is computerized and performed by the staff at the clinical research office at NRH.

### Intervention

The exercise intervention starts 5-10 months on ADT, one week after the pre-intervention assessment (Figure 3). The intervention is performed at the Norwegian School of Sport Sciences (NSSS) in Oslo.

The EG follows a modified resistance exercise program originally tested by Segal et al. [44]. Compared to the original program the intensity (training load) and duration are increased in order to potentially increase the effect of the strength training on LBM/body composition (Table 1). The patients perform three strength training sessions per week. Two of these sessions are performed under supervision by qualified instructors, to ensure safety, technique and progression in training load, with a maximum of three patients per instructor. The mid-week session, without the instructor, is performed at moderate intensity alone or together with other trial participants at NSSS. In each training set, the patient documents the training load and rate of exhaustion in an exercise log.

**Table 1:** The strength training program

Week	1. Session: heavy intensity	2. Session: moderate intensity	3. Session: heavy intensity
	Monday	Wednesday	Friday
**1 and 2**	2 × 10 sub maximal resistance	2 × 10 sub maximal resistance	2 × 10 sub maximal resistance
	Focus on correct technique	Focus on correct technique	Focus on correct technique
**3 to 6**	2 × 10 RM leg exercises	2 × 10 reps. leg exercises	3 × 6 RM leg exercises
	1 × 10 RM upper body	2 × 10 reps. upper body	2 × 6 RM upper body
		(resistance: 90% of 10 RM)	
**7 to 12**	3 × 10 RM leg exercises	2 × 10 reps. leg exercises	3 × 6 RM leg exercises
	2 × 10 RM upper body	2 × 10 reps. upper body	2 × 6 RM upper body
		(resistance: 90% of 10 RM)	
**13 to 16**	3 × 10 RM leg exercises	3 × 10 reps. leg exercises	3 × 6 RM leg exercises
	3 × 10 RM upper body	3 × 10 reps. upper body	3 × 6 RM upper body
		(resistance: 90% of 10 RM)	

*RM *- repetitions maximum, reps - repetitions

Each session consists of one to three sets of nine strength training exercises, performed at an intensity of 6 or 10 repetitions maximum (6-10 RM: the load that induces technique failure in 6 or 10 repetitions). The two first weeks of the program are considered to represent familiarization to the exercise protocol for the patients, and are performed at a light load (40-50% of one repetition maximum (1 RM)) in sets of 10 repetitions. The exercises performed are: smith machine squat, leg press, standing calf raises, knee flexion, knee extension, chest press, seated rows, shoulder press and preacher biceps curl (training equipment provided by Technogym, Italia).

Prior to the strength training session the patients perform 10 minutes of warm up on an exercise ergometer. In addition, the patients complete a sub maximal set of 10 repetitions as a specific warm-up in the squat exercise prior to every training session. After the two first weeks the patients are instructed to gradually increase the training load, in order to perform the exercise with the highest load possible during the prescribed number of repetitions per set on the two sessions with heavy intensity. From experience, this means that they increase the resistance by 2-5% per week through the 16 weeks period.

Patients in the CG are encouraged to maintain their habitual activity level and not start strength training. In order to increase the participation rate and reduce the dropout rate, the patients in the CG are offered the exercise intervention after the post-intervention assessment.

### Outcomes and assessments

Both clinical and muscle cellular outcomes are collected before the intervention (pre-intervention assessments) and after the intervention (post-intervention assessments) (Figure 3). All outcomes, specific variables and assessments in the PEPC trial are listed in Additional file 3.

#### Clinical outcomes

##### Body composition

LBM, BMD and fat mass are measured by dual-energy X-ray absorptiometry (DXA) using a Hologic multiple detector, fan-beam bone densitometer (Discovery QDR series). LBM is measured in arms, legs, trunk and total body. Changes in upper and lower body LBM are investigated separately because of differences in androgen sensitivity in leg muscles compared to neck, chest and shoulder muscles [45]. Body weight is measured by a digital platform scale and height by a stadiometer and body mass index (weight/(height)^2^) is calculated.

##### Serological outcomes

Fasting blood tests are taken between 8:00 am and 9:00 am. The tests analyzed are listed in Additional file 3. A biobank for frozen serum and full blood (EDTA) is established.

##### Physical functioning

Muscle strength is measured by 1 RM test, sit-to-stand test and stair-climbing test [46] and cardio-respiratory fitness is measured by Shuttle Walk test.

To secure validity of the physical tests, all patients undergo a session of familiarization to the actual tests 3-4 days prior to the pre- and post intervention assessments. Both sessions are performed based on the same guidelines, but after the familiarization session the load of each exercise is adjusted to match the expected 1 RM. Additional description of the physical functioning assessments is provided in Additional file 4.

##### Psycho-social functioning

Mental health are self-rated by the Hospital Anxiety and Depression scale (HADS) [47], fatigue is assessed by the Norwegian version of Fatigue Questionnaire (FQ) [48] and HRQOL by The European Organization and Treatment of Cancer Quality of Life Questionnaire (EORTC QLQ C-30) [49]. Additional description of the psycho-social functioning assessments is provided in Additional file 4.

#### Muscle cellular outcomes

Muscle biopsies are obtained from approximately half of the patients included in the study. Patients not willing to undergo biopsy are still eligible for trial participation.

With the patients in a supine position, a 6 mm Pelomi-needle (Albertslund, Denmark) with manual suction is used to obtain muscle samples (≈ 200 mg), under local anaesthesia (Xylocain^® ^adrenaline, 10 mg·ml^-1 ^+ 5 μg·ml^-1^, AstraZeneca, Södertälje, Sweden). Before the intervention the biopsy is obtained from the mid-section of the right vastus lateralis, and after the intervention the biopsy is obtained 3 cm proximal to the pre-intervention biopsy.

##### Muscle fibre size and regulators of muscle fibre size

*Muscle fiber size*, measured as muscle fiber cross sectional area, represents the primary muscle cellular outcome. Secondary muscle cellular outcomes reflecting *regulators of muscle fibre size *are a) number of myonuclei per muscle fiber b) number of satellite cells per muscle fiber, c) number of satellite cells and myonuclei positive for androgen receptors and d) proteins involved in muscle protein degradation (muscle breakdown); Forkhead Box Protein O (FOXO), Ubiquitin ligase E2 and Myostatin and muscle hypertrophy; androgen receptors and growth factors such as Insulin like growth factor 1 (IGF1) and Mechano growth factor (MGF).

Muscle fibre cross sectional area and regulators of muscle fibre size are analysed by immunohistochemistry on cross sections of muscle biopsies and by western blots and enzyme-linked immunosorbent assay (ELISA) in muscle homogenate.

Muscle fibre cross sectional area will be measured by cutting transverse serial sections of the muscle biopsy (8 μm thick) with a cryostat microtome (Microm, Germany) at -22°C and mounted on glass slides. Serial sections are immunohistochemically stained for fibre types (type I and type II) (used to measure muscle fibre cross sectional area), number of satellite cells, number of myonuclei and number of satellite cells and myonuclei positive for androgen receptors. Muscle fibre cross sectional area is measured for the different fibre types separately. An image of a satellite cell and basal lamina staining on a cross section from a muscle biopsy is shown in Additional file 1.

##### Regulators of muscle fibre function

Regulators of muscle fibre function studied in this project are proteins involved in the protection against cellular stress; heat shock proteins (HSP) 27 and HSP 70), as well as enzymes involved in mitochondrial function; Cytochrome C Oxidase 4 (Cox 4), Hsp 60 and Citrate synthase. These regulators are measured by western blots and ELISA.

#### Feasibility

The feasibility of the PEPC trial is investigated by examining the eligibility, compliance, attrition and safety among those considered for inclusion and those participating. This involves registration of a) the number of eligible patients among all PC patients receiving radiotherapy combined with ADT, b) the number of patients willing to participate among the eligible patients and c) compliance and adherence to the intervention programs and reasons for missing exercise sessions and discontinuation

#### Background variables

The patients provide information about partnership, number and age of children living at home, education, work and sick leave by filling out a questionnaire. Information about medical situation as time points for treatment, stage of disease and comorbidity are collected from the medical record. Past illnesses and other medical problems are also reported in the questionnaire.

##### Lifestyle outcomes

The level of physical exercise is assessed by a modified Norwegian version of the Leisure Score Index (LSI) from the Godin Leisure-Time Exercise Questionnaire (GLTEQ) [50]. Dietary habits are assessed by a modified version of the SmartDiet - a short food questionnaire [51]. Smoke- and snuff habits are measured by two single questions; Do you smoke? "Yes daily", "Yes, now and then", "I smoked earlier but I have stopped" and "No, I have never smoked" and Do you take snuff? "Yes, daily", "Yes, now and then", "I snuffed earlier but I have stopped" and "No, I have never snuffed".

### Sample size and statistical considerations

LBM is the primary endpoint of the study. Sample size calculation is based on comparing the clinically relevant difference in change before and after the 16 weeks period in LBM between the EG and CG. To detect a 3 kilograms difference in change between the groups, and assuming a standard deviation (SD) of 3 kilograms, 22 patients are needed in each group with a significance level (two sided) of 5% and a power of 90%. Due to dropouts we plan to include 30 patients in each group. According to previous studies, this number should also be sufficient to detect differences in the cellular muscle outcomes (Sinha-Hikim et al., 2006).

Changes in outcome variables from start of intervention will be compared between the EG and CG groups by analysis of covariance using the baseline measurement as a covariate. P-values below 5% will be regarded as statistically significant. If obvious deviations from normal distributions are detected, changes in outcome will be compared between groups by Wilcoxon-Mann-Whitney test. The primary analysis population will be the intention to treat population using last observation carried forward to impute any missing values. In addition a per-protocol analysis including only patients with no missing observations of the variable of interest will be performed. Should imbalances in important variables be detected sensitivity analyses will also be added including these as covariates in the model.

## Discussion

Previous research has examined the effect of physical exercise on clinical outcomes such as body composition, physical function, mental health, fatigue and quality of life in PC patients [3-5]. Still research on the effect of physical exercise in this patients group is less extensive than in other cancer groups, such as for example patients with breast cancer. Two of the most pronounced clinical side effects of ADT are the negative effect on muscle mass and muscle strength. However, studies investigating the effects of ADT and exercise at the muscle cell level are still lacking. Expanded knowledge in this field among PC patients is therefore required.

The clinically indicated ADT in PC patients enables the study of testosterone's role in regulation of muscle mass during strength training. The main question is whether normal adaptations to strength training are disturbed when the level of testosterone is below 1 nmol/L (castration level); e.g. is the activation of satellite cells and hypertrophic effect of strength training blunted in these patients? Furthermore, by investigating important factors for the regulation of muscle mass and muscle function, the cellular effects of ADT on muscle tissue will be elucidated. Currently, three other ongoing trials are investigating the effect of exercise on muscular outcomes assessed by muscle biopsies in patients with breast, lung and testicular cancer receiving chemotherapy [52-54]. However, the effects of high-load strength training on muscular adaptations in PC patients during ADT have not previously been investigated.

Compared to previous studies in PC patients the intensity and/or duration in our strength training protocol is somewhat increased [44,55,56]. The rationale for choosing a strenuous strength training protocol is the fact that the effect on total muscle mass has been moderate or absent in previous studies on PC patients. One possible explanation for negligible effects on muscle mass in previous studies may be that the stimuli for muscle hypertrophy have been too low. In healthy men, it is suggested that a training intensity of 75-80% of 1 RM (6-12 RM sets) combined with multiple sets in each exercise (starting from 1-2 and progressing to 3-6 sets per exercise), give optimal stimuli for muscle hypertrophy when performed 2-3 times per week [57]. Consequently, in order to maximize the stimuli for muscle hypertrophy we chose to implement this strength training protocol in our patients. Importantly the training protocol should still be tolerable and safe.

Positive findings from the PEPC trial will help in the construction of more effective training protocols to counteract the negative effects of ADT on muscle tissue, BMD and physical function. More importantly, the results on the cellular effects of ADT in muscle tissue will provide novel understandings of muscle mass regulation by testosterone. Such knowledge will improve our understanding on how aging in general and reduced testosterone production during ADT specifically, induces loss of muscle mass (sarcopenia). In turn this knowledge can be used to establish more effective strategies against sarcopenia in both healthy elderly and in patient populations at risk. New strategies could include both new medical treatments and training strategies focused to overcome the cellular changes inducing sarcopenia.

### Summary

Knowledge of the effect of exercise on clinical outcomes such as physical functioning and quality of life outcomes has increased over the last decades. Studies testing aerobic exercise in breast cancer patients are most frequent. Studies in other cancer groups testing the effects of strength training program are still lacking. The PEPC trial focuses on the effect on a high-load strength training program on both clinical and muscle cellular outcomes in PC patients during ADT. As far as we know this is the first study having a muscular cellular outcome in these patients. By testing a high-load strength training program the study will provide new knowledge on optimal training programs in PC patients.

## Competing interests

The authors declare that they have no competing interests.

## Authors' contributions

All authors read and approved the final manuscript. LT: provided substantial contribution to the conception and design of the study and drafted the manuscript, TSN: provided substantial contribution to the conception and design of the study and drafted the manuscript, TR: provided substantial contribution to the conception and design of the study and helped to draft the manuscript, KSC: provided substantial contribution to the conception and design of the study and helped to draft the manuscript, ES: provided substantial contribution to the design of the study and helped to draft the manuscript and SDF: provided substantial contribution to the conception and design of the study and helped to draft the manuscript.

## Endnote

^1^The term muscle fiber (muscle cell) originates from the embryonic formation of mature muscle cells where mononuclear myoblasts fuse to the giant multinuclear muscle cells.

## Pre-publication history

The pre-publication history for this paper can be accessed here:

http://www.biomedcentral.com/1471-2407/12/123/prepub

## Supplementary Material

Additional file 1 **An image of a satellite cell and basal lamina staining on a cross section from a muscle biopsy**. Click here for file

Additional file 2 **Inclusion and exclusion criteria**. Click here for file

Additional file 3 **Outcomes, specific variables and assessments**. Click here for file

Additional file 4 **Descriptions of assessments**. Click here for file
