# Supplementary material for: A randomized controlled trial on the effectiveness of strength training on clinical and muscle cellular outcomes in patients with prostate cancer during androgen deprivation therapy: rationale and design
Source: BMC Cancer. 2012 Mar 29;12:123. doi: 10.1186/1471-2407-12-123 (PMC3342229; doi:10.1186/1471-2407-12-123)
Supplement: Additional file 1 — An image of a satellite cell and basal lamina staining on a cross section from a muscle biopsy. [file 1471-2407-12-123-S1.PDF]

Additional file 4 – An image of a satellite cell and basal lamina staining on a cross section from a muscle biopsy

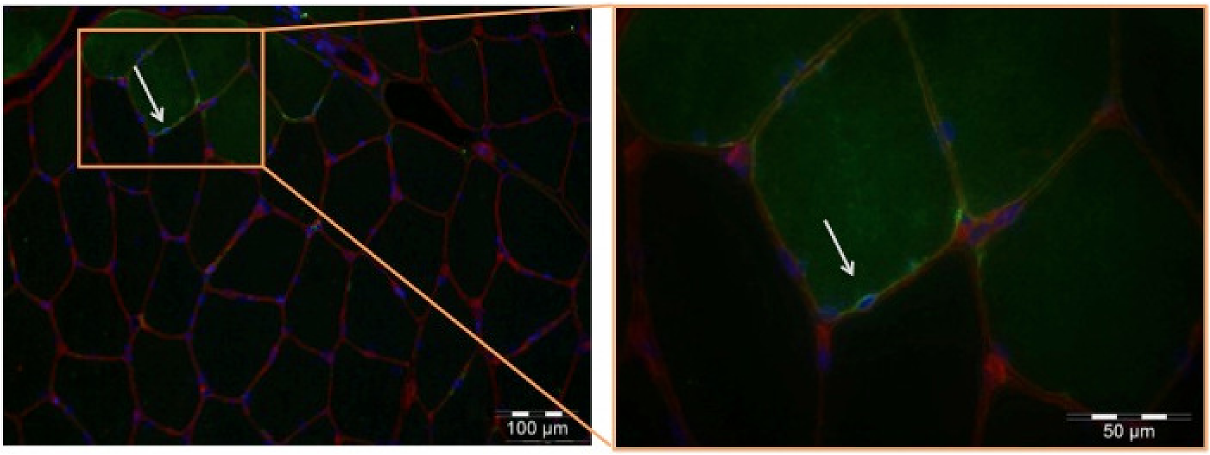

Thin cross section (8 µm) of a muscle biopsy with antibody staining towards the basal lamina protein ***laminin*** (red). ***Laminin*** staining is used to visualize the circumference of the muscle fiber, which in turn is measured and the muscle fiber cross sectional area is calculated.

An antibody against a protein in the membrane of the satellite cell, ***neural adhesion molecule*** (Ncam) (green), is used to identify satellite cells. Ring-like Ncam staining, inside the laminin staining, and with a nucleus inside, is manually counted as a satellite cell.

DAPI binds to DNA and stains all nuclei (blue). An antibody against the plasma membrane ***protein dystrophin*** is used to stain the plasma membrane(not shown). DAPI staining inside the plasma membrane (dystrophin ring) is manually counted as a myonucleus.
