# Supplementary material for: A randomized controlled trial on the effectiveness of strength training on clinical and muscle cellular outcomes in patients with prostate cancer during androgen deprivation therapy: rationale and design
Source: BMC Cancer. 2012 Mar 29;12:123. doi: 10.1186/1471-2407-12-123 (PMC3342229; doi:10.1186/1471-2407-12-123)
Supplement: Additional file 2 — Inclusion and exclusion criteria. [file 1471-2407-12-123-S2.PDF]

Additional file 1: Inclusion and exclusion criteria

Inclusion / Randomization Form

Patient No.: ☐☐☐ **Patient's Initials:** ☐☐☐ **Patient's Date of Birth:** ☐☐.☐☐.☐☐☐☐ (day, month, year)

Patient's Name: ☐

☐ ***Inclusion*** ☐ ***Randomization***

INCLUSION CRITERIA

1. Newly diagnosed locally advanced prostate cancer (clinical stage T2 and T3)	☐ Yes	☐ No
2. Referred to RH and UUH for radiotherapy	☐ Yes	☐ No
3. < 75 years of age	☐ Yes	☐ No
4. Capable of reading and writing Norwegian	☐ Yes	☐ No
5. Treating oncologist has approved the subjects' participation	☐ Yes	☐ No
6. Lives within approximately 1 hour from Oslo by car or public transportation	☐ Yes	☐ No
7. Written informed consent received	☐ Yes	☐ No

EXCLUSION CRITERIA

1. Routine resistance training with manuals	☐ Yes	☐ No
2. Medication for osteoporosis (i.e. bisphosphonates)	☐ Yes	☐ No
3. Conditions that contraindicate exercise without adjusted actions:		
a) unregulated hypertension (>160/95 mmHG)	☐ Yes	☐ No
b) uncontrolled cong. heart failure (NYHA class > II)	☐ Yes	☐ No
c) unstable angina pectoris	☐ Yes	☐ No
d) recent myocardial infarction (last 6 months)	☐ Yes	☐ No
e) cardiac arrhythmia	☐ Yes	☐ No
f) chronic obstructive pulmonary disease	☐ Yes	☐ No
g) substantial lung, pleural or pericardial disease	☐ Yes	☐ No
h) severe asthma	☐ Yes	☐ No
i) recent stroke	☐ Yes	☐ No
j) epilspsy	☐ Yes	☐ No
k) insulin dependent diabetes mellitus	☐ Yes	☐ No
l) unstable bone lesions with high risk of fractures	☐ Yes	☐ No
4. Mentally incompetent conditions:		
a) severe anxiety or depression	☐ Yes	☐ No
b) dementia	☐ Yes	☐ No
c) known alcoholism or other abuse (liver test)	☐ Yes	☐ No
d) mentally retarded	☐ Yes	☐ No
5. Conditions complicating ability to participate in a supervised training program:		
a) uncontrolled pain	☐ Yes	☐ No
b) severe arthritis	☐ Yes	☐ No
c) scheduled hip or knee replacement	☐ Yes	☐ No
d) pathologic fractures last 6 months	☐ Yes	☐ No
e) amputation	☐ Yes	☐ No
f) walker or wheelchair use	☐ Yes	☐ No

Clinician's Signature: ____________________ Date: ☐☐.☐☐.☐☐☐☐ (day, month, year)

RH – The Norwegian Radium Hospital
UUH – Ullevaal University Hospital
