# Supplementary material for: A randomized controlled trial on the effectiveness of strength training on clinical and muscle cellular outcomes in patients with prostate cancer during androgen deprivation therapy: rationale and design
Source: BMC Cancer. 2012 Mar 29;12:123. doi: 10.1186/1471-2407-12-123 (PMC3342229; doi:10.1186/1471-2407-12-123)
Supplement: Additional file 3 — Outcomes, specific variables and assessments. [file 1471-2407-12-123-S3.PDF]

Additional file 2: Outcomes, specific variables and assessments

**Outcomes**	**Specific variables**	**Assessments**
Clinical outcomes		
Body composition		
	Lean Body Mass (LBM) (primary outcome)	DXA
	Bone Mineral Density (BMD)	DXA
	Fat mass	DXA
	Body Mass Index	Weight and height
Serological outcomes		
	Hemoglobin, Hematocrit, Leucocytes, Thrombocytes, Sodium, Potassium, Calcium, ALAT, Total Cholesterol, LDL Cholesterol, HDL Cholesterol, Triglyceride, Testosterone, Estrogen, LH, FSH, SHBG, Albumin, Total Protein, CRP, PSA	
Physical functioning		
	Muscle strength	1 RM, Sit-to-stand test and Stairs-climbing test
	Cardio-respiratory fitness	Shuttle walk test
Psycho-social functioning		
	Mental health	HADS
	Fatigue	FQ
	HRQOL	EORTC QLQ C-30

Muscle cellular outcomes		
Muscle fiber size	Muscle fiber cross sectional area (primary cellular outcome)	Cross sections of muscle biopsies

Regulators of muscle fiber size		
Number of myonuclei per muscle fiber		Cross sections of muscle biopsies
Number of satellite cells per muscle fiber		Cross sections of muscle biopsies
Number of satellite cells and myonuclei positive for androgen receptors		Cross sections of muscle biopsies
Proteins involved in muscle protein degradation (muscle breakdown)	FOXO, Ubiquitin ligase E2	Muscle homogenate (western blot)
	Myostatin	Muscle homogenate (ELISA), mRNA
Proteins involved in muscle hypertrophy	Androgen receptors	Muscle homogenate (western blot)
	Growth factors (IGF1 and MGF)	Muscle homogenate (ELISA), mRNA

Regulators of muscle fiber function:		
Proteins involved in cellular stress (stress proteins)	Hsp 27, 60 and 70	Muscle homogenate (western blot and ELISA)
Proteins involved in mitochondrial function	COX 4 and Citrate synthase (mitochondrial enzymes)	Muscle homogenate (western blot and ELISA)
Background variables		
	Demographic and medical variables<br>Level of physical exercise<br>Dietary habits<br><br>Smoke- and snuff habits	 GLTEQ<br>Smart diet – a short food questionnaire<br>Smoke and snuff questions

DXA - Dual-energy X-ray Absorptiometry, LDL - Low Density Lipoprotein, HDL - High Density Lipoprotein, LH - Luteinizing Hormone, FSH - Follicle Stimulating Hormone, SHBG - Sex Hormone-Binding Globulin, CRP - C-Reactive Protein, PSA - Prostate Specific Antigen, HADS - Hospital Anxiety and Depression Scale, FQ - Fatigue Questionnaire, HRQOL – Health-Related Quality Of Life, EORTC QLQ C-30 - The European Organization for Research and Treatment of Cancer Quality of Life Questionnaire C-30, FOXO – Forkhead Box Protein O, ELISA - enzyme-linked immunosorbent assay , IGF1 – Insulin growth factor 1, MGF - Mechano growth factor, Hsp - Heat Shock Proteins, GLTEQ - The Godin Leisure-Time Exercise Questionnaire, COX 4 – Cytochrome C Oxidase 4
