# Supplementary material for: A randomized controlled trial on the effectiveness of strength training on clinical and muscle cellular outcomes in patients with prostate cancer during androgen deprivation therapy: rationale and design
Source: BMC Cancer. 2012 Mar 29;12:123. doi: 10.1186/1471-2407-12-123 (PMC3342229; doi:10.1186/1471-2407-12-123)
Supplement: Additional file 4 — Descriptions of assessments. [file 1471-2407-12-123-S4.PDF]

Additional file 3 - Description of assessments

Physical functioning

Muscle strength

The *1 RM tests* are performed in two leg exercises (leg press and knee extension) and two upper body exercises (chest press and shoulder press). After warm-up, consisting 10 minutes on an exercise ergometer and 4 sets with gradually increasing load (40-75-85-95% of expected 1RM) and decreasing number of repetitions (10-6-3-1) subjects performs the first 1RM attempt. After each successful attempt, the load is increased. The aim is to find the 1RM within maximum five attempts. The rest period between each attempt is 2-3 minutes. In the *sit-to-stand test* the patients sit on a 46-cm high hardback chair without armrest, with their arms folded. One sit-stand cycle is completed when the patient rise to full extension in the knee joint, and sit down leaning the lower back to the back support. The patients are instructed to complete as many cycles as they are able to in 30 seconds [46]. In the *stair-climbing test* the patients climb a flight of stairs (20 stairs per flight, 16-cm rise per stair) as rapidly as they can safely manage without use of the handrails. The patients are timed from bottom of the stairs to the top by photocells (Browning timing system). After two successful attempts the patients are loaded with 20 kg, by wearing a 10 kg weight west and carrying two hand manuals of 5 kg each, and complete two more runs [46]

Cardio-respiratory fitness

In the scuttle walk test the patient walks back and forth on a 10 meters distance. The walking pace is given by a beep-signal provided by a CD-player. The “beep” indicates when the patient should turn and head back to the other side. The initial pace is set to 30 meters per minute (1.8 km/t), thereafter the intervals between signals are reduced every

minute leading the patient to increase their pace by 10 meters per minute. The maximal duration of the test is 12 minutes, resulting in a pace of 140 meters per minute (8.4 km/t). The test is over if the patient is not able to keep up with the pace, feels too exhausted to continue or completes the entire 12 minutes. The heart rate is measured and registered at the beginning and immediately after the test.

Psycho-social functioning

Mental health

HADS consists of 14 items (HADS-T), seven for depression (HADS-D) and seven for anxiety (HADS- A). Each item has four scoring alternatives ranging from zero (not present) to three (highly present). The sum scores of HADS-A and HADS-D range from zero to 21, respectively. HADS-defined anxiety disorder and depression are defined by an empirically derived cut-off score of $\geq$ eight on HADS-A and HADS-D. The total HADS score range from zero to 42, where scores from 16 to 42 are defined as cases of HADS-defined mental distress [93].

Fatigue

FQ contains 11 items reflecting total fatigue (TF). Seven of these items reflect physical fatigue (PF) and four items reflect mental fatigue (MF). Additionally, two items ask about the duration and extent of fatigue. Each item has four response choices with a Likert-scoring (0,1,2,3), for summarizing TF, PF and MF respectively. Higher scores imply more fatigue. Chronic fatigue is defined by using a dichotomized score (0,0,1,1). If the sum of the dichotomized scores is $\geq$ 4 and the duration of fatigue six months or longer the respondent is defined as chronic fatigued.

Health related quality of life

EORTC QLQ C-30 comprises five functional scales (physical, role, emotional, social and cognitive), three symptom scales (fatigue, pain and nausea/vomiting), six single items assessing additional symptoms commonly reported by cancer patients and two questions on patients' overall QOL and overall health condition, providing a global QOL score. These scales are transferred to a zero to 100 point scale, calculated by using the scoring manual provided by the EORTC [90]. For the functional scales and global QOL scales a higher score indicates better level of functioning, while increasing values on the symptom scales and single items indicate increased symptom load. We also use the PC module, focusing on special issues for patients with PC such as sexual function, incontinence and bowel function.
